# Microfluidic Devices for Drug Delivery Systems and Drug Screening

**DOI:** 10.3390/genes9020103

**Published:** 2018-02-16

**Authors:** Samar Damiati, Uday B. Kompella, Safa A. Damiati, Rimantas Kodzius

**Affiliations:** 1 Department of Biochemistry, Faculty of Science, King Abdulaziz University (KAU), Jeddah 21589, Saudi Arabia; 2 Department of Pharmaceutical Sciences, Ophthalmology, and Bioengineering, University of Colorado Anschutz Medical Campus, Aurora, CO 80045, USA; Uday.Kompella@ucdenver.edu; 3 Department of Pharmaceutics, Faculty of Pharmacy, King Abdulaziz University (KAU), Jeddah 21589, Saudi Arabia; smdamiati@kau.edu.sa; 4 Mathematics and Natural Sciences Department, The American University of Iraq, Sulaimani, Sulaymaniyah 46001, Iraq; 5 Materials Genome Institute, Shanghai University, Shanghai 200444, China; 6 Faculty of Medicine, Ludwig Maximilian University of Munich (LMU), 80539 Munich, Germany; 7 Faculty of Medicine, Technical University of Munich (TUM), 81675 Munich, Germany

**Keywords:** drug and gene delivery systems, in vitro drug screening, cell-on-a-chip, organ-on-a-chip, human-on-a-chip

## Abstract

Microfluidic devices present unique advantages for the development of efficient drug carrier particles, cell-free protein synthesis systems, and rapid techniques for direct drug screening. Compared to bulk methods, by efficiently controlling the geometries of the fabricated chip and the flow rates of multiphase fluids, microfluidic technology enables the generation of highly stable, uniform, monodispersed particles with higher encapsulation efficiency. Since the existing preclinical models are inefficient drug screens for predicting clinical outcomes, microfluidic platforms might offer a more rapid and cost-effective alternative. Compared to 2D cell culture systems and in vivo animal models, microfluidic 3D platforms mimic the in vivo cell systems in a simple, inexpensive manner, which allows high throughput and multiplexed drug screening at the cell, organ, and whole-body levels. In this review, the generation of appropriate drug or gene carriers including different particle types using different configurations of microfluidic devices is highlighted. Additionally, this paper discusses the emergence of fabricated microfluidic cell-free protein synthesis systems for potential use at point of care as well as cell-, organ-, and human-on-a-chip models as smart, sensitive, and reproducible platforms, allowing the investigation of the effects of drugs under conditions imitating the biological system.

## 1. Introduction

Development of novel drugs and new strategies for efficient delivery are attractive to enhance treatment outcomes by improving bioavailability and specificity of the therapeutic agent, while minimizing toxicity. Many conventional bulk methods to synthesize drug or gene delivery systems suffer from several drawbacks such as the need to use a large volume of valuable drugs or chemicals, the generation of polydisperse particles that affect the release profile, the limitation of generating carriers loaded with multiple therapeutic agents, and the difficulty associated with localizing drug delivery and investigating the therapeutic/toxic effects in vivo, which requires many animals [1,2,3,4]. 

Microfluidic or lab-on-a-chip (LOC) technology is attracting growing interest in recent years. It is based on the science that deals with the fabrication of microdevices with small channels and chambers and control of the flow behavior of small volume of fluids in micro-channels and micro-chambers, whose dimensions are in the range of tens to hundreds of micrometers [5]. Microfluidics is a multidisciplinary technology intersecting nanotechnology, biotechnology, biochemistry, physics, and engineering [6]. Microfluidic technologies developed in the early 1980s paved way for several promising platforms, offering many advantages such as reducing the consumption of reagents/samples to pico-liters; decreasing the reaction time to seconds; reducing waste generation; allowing rapid diffusion, mass and heat transfer; enabling the modeling of physiological conditions more accurately for 3D cell-culture and cell-based assays; enabling a continuous nutrient and oxygen supply; allowing the integration of multiple steps such as cell culture, cell capture, cell lysis, and mixing; and detection on the same device [7,8,9,10]. In addition to the ability of microfluidic devices to produce reproducible and homogeneous gene/drug micro- or nanocarriers of the desired size and shape, they present useful in situ platforms for drug screening in the form of cell-on-a-chip, organ-on-a-chip, and human-on-a-chip platforms to assess drug responses and partially replace animals in research. Integration of microfluidic components such as valves and pumps allows high-throughput and multi-plexed drug screening.

Several factors should be taken into consideration when designing microfluidic devices, such as the type of materials used to fabricate the device, the compatibility of these materials with the various solvent channel dimensions, the number of inlets, and the types of synthesizers and mixers [11]. Polydimethylsiloxane (PDMS) and poly(methyl methacrylate) (PMMA) are the most commonly used polymers to fabricate microfluidic devices by the soft-lithography technique [12,13]. These materials can be engineered to allow oxygen entry. The main disadvantage of these two polymers is their susceptibility to swelling when exposed to strong solvents such as acetone. Swelling of microfluidic channels affects fluid flow and uncontrolled carrier generation [14]. Alternative polymers that are chemically resistant to strong solvents include polytetrafluoroethylene (PTFE) and cyclic olefin copolymer (COC), which are used to fabricate microfluidic devices by the hot-embossing technique [15,16]. 

Production susceptibility and efficacy of generated drug/gene carriers rely on microfluidic fabricators such as diffusion-based mixer, droplet generator, chaotic mixer, and automated microfluidic system. The microfluidic diffusion-based mixer is one of the most commonly used synthesizers. It is composed of multiple inlets and one outlet. In this system, the reactions between carrier materials and molecules are controlled by the channel length and flow rate. To increase the diffusive boundary area of the samples by at least two orders of magnitude, incorporation of the herringbone pattern enhances the rapid and reproducible generation of drug delivery systems. However, mixing methods that rely on the diffusion between separated flow streams under a continuous flow process in the boundary may result in large amounts of unreacted materials, which may increase the cytotoxicity and lowering of the rate of drug transport. Hence, droplet generation techniques are a promising alternative solution that enable reactions to occur within the droplets independently. In this system, the reaction rate and size, the total volume, and the ratio between reacted molecules in the microchannel can be controlled precisely while avoiding contamination issues [17,18].

In this review, the strategies and applications of microfluidic technology for drug and gene carrier generation and drug screening platforms are discussed. Fabrication methods of drug/gene-loaded particles, including self-assembly, droplet, and non-spherical carriers, are highlighted to form different particle types. Moreover, microfluidic platforms that act as a supporting matrix for 3D cell culture at the cell, organ, and human levels are reviewed, as well as in vitro synthesis of proteins on the microfluidic chip. These devices and their potential applications are introduced in this review. 

## 2. Self-Assembled Drug Delivery System

**Figure 1:**
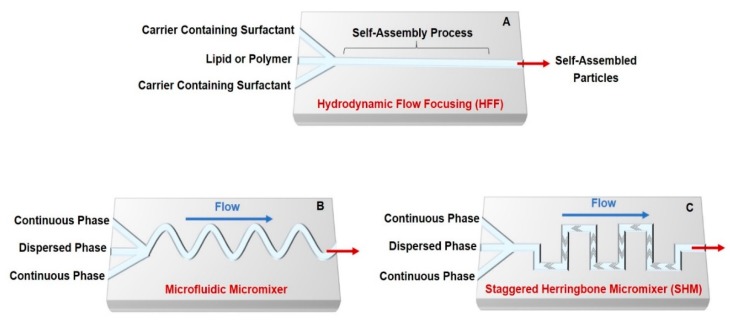
Microfluidic platforms for generation of self-assembled drug and gene carriers: (**A**) schematic of a simple hydrodynamic device with HFF; (**B**) schematic of a simple microfluidic mixing chamber; and (**C**) schematic of SHM for chaotic mixing Figure modified from References [19,28,29].

Nano- and micro-size self-assembling gene and drug delivery systems have been generated in microfluidic systems that enable the formation of reproducible and homogeneous assemblies. To generate self-assembling drug delivery systems in microfluidics, two or multiple streams of several reagents are interfaced, and the carriers are generated at the interfacial layer (Figure 1). In most PDMS microfluidic chips, the molded channels are rectangular, which helps to apply the concept of hydrodynamic diameter (D_h_). The following formula can calculate the D_h_:

(1) $$D_{h}=\frac{4xA}{P_{wet}}$$

where *A* is the cross-section area of flow-through and *P_wet_* is the wetted perimeter where the perimeter of the channel is in direct contact with the flow. The calculation of *D*_h_ also helps to estimate the flow through irregular channel shapes such as channels with a circular cross-section. When the channel is completely filled, the *P_wet_* and *A* values are the diameter and cross-section area of the channel, respectively [19]. 

Some platforms suitable for preparing self-assembled particulate drug delivery systems include hydrodynamic flow focusing (HFF), microfluidic mixer in conjunction with staggered Herringbone micromixer (SHM), or microfluidic mixer in conjunction with SHM. Self-assembled carriers are commonly generated through HFF by controlling the mixing rates between fluid streams based on the microchannel shapes, flow rates, and diffusion coefficients of different miscible streams (Figure 1A). Setting the flow rate ratios (FRR) of water and a solvent containing polymers or lipids precisely enhances the self-assembly reaction and controls the degree of mixing within the microfluidic channel [17,18,20,21,22,23,24,25,26,27]. Increasing the ratio of the flow rate of solvent to that of water induces the slow mixing and generation of large nanoparticles. 

In the HFF method, a core of carrier fluid containing the surfactant mixer is concentrated by surrounding streams of a miscible solution in the microchannel. After precise formation of self-assembled lipid or polymeric particles by microfluidic mixing, encapsulation or chemical coupling of active molecules is loaded into the synthesized particles. The HFF method usually produces self-assembled drug delivery systems smaller than 1 µm, which might allow better delivery across the physiological barriers. Moreover, in the microfluidic HFF system, the narrow width of the core stream offers fast mixing due to a small diffusion length scale. The diffusive mixing time (T_mix_) can control the particle size distribution and generation rate, which can be calculated according to the following formula:

(2) $$\tau_{mix\;}=\;\frac{\omega_{f}^{\;\;2}\;}{4D}\;\approx \;\frac{\omega^{2}\;1}{9D\;{\left(1\;+\;FRR\right)}^{2}}$$

where *ω_f_* is the focused stream width, *ω* is the channel width, *D* is the diffusivity of the fluid in the core stream, and *FRR* is the flow rate ratio of the core stream flow rate to the total flow rate of the surrounding streams. Increasing the flow ratio, *R*, in a fixed geometry can enhance the production rate and increase the average diameter of synthesized nanoparticles [27,30,31]. 

To ensure rapid, subsequent self-assembled droplet generation in microfluidic devices, a micromixer has been reported for the development of micro- and nanoparticles (Figure 1B) [32]. Indeed, the efficiency of many biosensors and the possibility of investigating reaction kinetics with good time resolution depend on controlled mixing. In many microfluidic procedures, the integrated micromixer is an essential tool for appropriate functionality in a broad range of applications. Micromixers are mainly categorized as active and passive mixers. In active systems, an external force such as mechanical vibration or pneumatics is required to improve mixing efficiency. This system is difficult to control and to combine with other microfluidic components [33]. In contrast, a passive mixer is easy to manufacture and integrate with other microfluidic components. This system allows the interfaced streams to be mixed by introducing surface micro-architecture or by a sudden change in the flow configuration without any requirement for external agitation [33,34,35]. For example, Belliveau et al., [36] designed a microfluidic device with a passive mixer that offers higher throughput than HFF (Figure 1C). This SHM generates lipid nanoparticles (LNPs) encapsulating small interfering RNA (siRNA) with a size range of 20–100 nm, low polydispersity, and high encapsulation efficiency. The developed device shows the rapid synthesis of gene-containing carriers with a good gene silencing efficiency compared to other formulation processes. Another example shows the ability of the herringbone mixer to generate LNPs of controlled size consisting of 1-palmitoyl-2-oleoyl-sn-glycerol-3-phosphocholine (POPC), cholesterol and triglyceride triolein [37]. Staggered Herringbone micromixer provides a high mixing rate and allows milli-second mixing of aqueous and ethanol-containing lipid streams at high FRR to synthesize self-assembled bottom-up LNPs [37]. 

Another microfluidics system is simple diffusion-based, which offers an extra advantage unavailable in the HFF and micromixer systems, namely, the ability to generate multilayer carriers to deliver multiple factors. This system allows subsequent reaction steps by the injection of multiple flow streams into a single channel [38,39]. Lipoplex nanoparticles encapsulating deoxyoligonucleotide (DON) formed with a 5-inlet microfluidic device exhibit better delivery or cellular uptake in human leukemia cells than particles synthesized by conventional bulk mixing methods [40]. Tran et al., [41] reported highly efficient production with a short reaction time and encapsulation of amphiphilic heparin-folate-retinoic acid (HFR) bio-conjugates. Although the self-assembled nanoparticles produced had relatively low loading efficiency, those carriers exhibited high selectivity and efficiently delivered the drug to cancer cells that are highly expressed folate receptors with superior cellular uptake compared to particles generated by a bulk reaction [41]. 

Each drug carrier has advantages and disadvantages. Self-assembled drug delivery system shows the advantages of high drug content, controlled release, and potentially macrophage-specific distribution. In contrast, this system is not typically able to incorporate amphiphilic drugs, and, therefore, these agent structures need to be modified chemically before manufacturing self-assembled drug delivery system.

## 3. Droplet-Based Carriers

**Figure 2:**
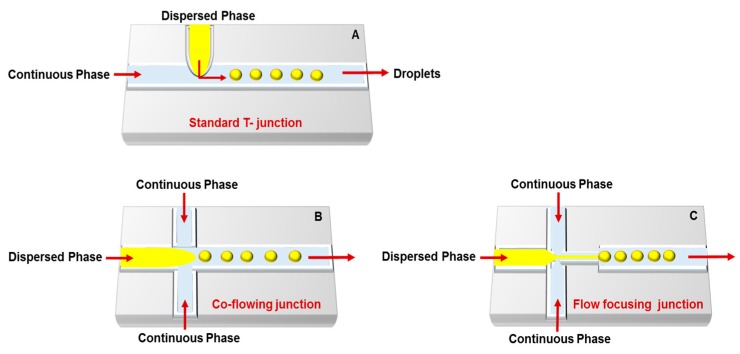
Droplets microfluidic platforms for generation of drug and gene carriers: (**A**) T-junction; (**B**) co-flowing junction; and (**C**) flow-focusing junction. Dispersed phase consists of divided droplets that suspended in the continuous phase Figure modified from References [44,45].

Droplet-based microfluidics is the most used carrier production method; it synthesizes mono-dispersed and size-controlled nano- and micro-particles. This technique enables biomolecule encapsulation into discrete droplets and performs analysis with these generated units. The droplet generation strategies include: (i) hydrodynamics (T- and Y-junction, flow-focusing, and co-flowing); (ii) pneumatic pressure, which uses gas pressure as a shear force and a driving force to form droplets; (iii) optical techniques that use optical force to form the particles; and (iv) electrical techniques (dielectrophoresis (DEP) and electrowetting on dielectric (EWOD)) [42,43]. The most important microfluidic configurations for droplet generation are as follows (Figure 2).

**T-junction:** The simplest and most used microfluidic geometry. In standard geometry, the orthogonal channel contains the dispersed phase that intersects the main channel, which filled with the continuous phase and droplets synthesized at the channel intersection. Hydrophilic channels can generate oil-in-water (O/W) or double water-in-oil-in-water (W/O/W) emulsions, while hydrophobic channels generate water-in-oil (W/O) or oil-in-water-in-oil (O/W/O) emulsions [46,47]. Indeed, some T-junction devices can prepare O/W and W/O emulsions by careful choice of surfactants and addition to the aqueous or oil phase [44,45,48]. Three central regimes of droplet formation in T-junctions are distinguished based on the capillary number: dripping, jetting and squeezing [49]. The droplet size in this system can be controlled either actively or passively. Active control can be regulated using external actuation such as integrated micro-heaters, pneumatically or magnetically actuated micro-valves, or a controllable moving wall structure, while passive control can be modulated by controlling the flow rate [14,50,51,52]. 

**Co-flowing junction:** Dripping and jetting are the two main types of co-flowing devices. Several factors affect the formation of droplets in co-flow systems, such as fluid velocities, surface tension, viscosities, densities, and channel geometries. Co-flow in microfluidic devices occurs when the dispersed phase is injected into a small capillary centered inside a larger-diameter channel with a continuous phase that flows in parallel to the dispersed phase. The continuous phase surrounds the dispersed phase, which makes the viscous shear force stronger as the diameter of the dispersed phase increases. At low flow rates of both the continuous and dispersed fluids, dripping is the predominant regime, resulting in the formation of spherical droplets. At high flow rates, the jetting regime is predominant, and droplets form by breaking a thin stream of dispersed phase farther downstream due to convective instabilities [44,53].

**Flow focusing junction:** Planar microfluidic flow-focusing devices (MFFDs) are the most commonly used flow focusing systems. In these devices, the middle channel contains the dispersed phase, while the two outside channels contain the continuous phase [54]. Both continuous and dispersed phases are forced through the orifice located downstream of the three channels. In addition to the extended pressure and shear stress generated by the continuous phase in the symmetric channels, the flow of the dispersed phase into open channel forces the dispersed phase through a small orifice, resulting in filling the larger channel with the continuous phase and breaking the dispersed phase inside or downstream of the orifice. The flow pattern can be planar or cylindrical based on the channel and orifice geometries. However, droplet size, velocity, and frequency can be controlled by controlling the flow rates and inlet pressures and altering the phase viscosities and orifice size [44,55,56,57].

Similar to self-assembled systems, droplet microfluidic techniques include active and passive methods that allow generation of nanoliter- to femtoliter-sized discrete droplets in a microchannel by combing shear stress with the interfacial tension between the continuous aqueous stream and the immiscible carrier phase. The generation of active droplets requires acoustic, electrical, pneumatic, magnetic, or thermal forces incorporated into the microfluidic system, whereas passive droplets can be generated by co-flowing, cross-flowing, flow-focusing, T-junction, and step emulsification [58,59,60,61,62]. Although existing methods are more difficult to fabricate in miniature format, they show more flexibility in droplet manipulation than passive [54,63,64,65,66,67,68]. In droplet-based microfluidics, the immiscible fluids are brought together just before droplet generation in a co-flow channel. This rapid mixing in separated droplets overcomes diffusion limit mixing and fluid dispersion [69]. Hence, efficient mixing in a microfluidic device is the key to generating well-defined monodispersed particles. The fast mixing in the droplets is due to the high surface area-to-volume ratio and shorter diffusion distances between molecules. Encapsulation of the target analyte into the droplets offers several advantages, such as excluding sample loss on the surface wall by preventing the contact between the sample and the droplet wall and preventing analyte leakage and cross-contamination between droplets. Moreover, in contrast to continuous microfluidics, droplet-based systems overcome the complicated fluidic control, do not require separated channels for each sample, and minimize the dilution and contamination problems [70,71].

The two main types of droplet-based techniques are digital and segmented-flow microfluidic systems. Digital microfluidic devices are fabricated to separate nanoliter to microliter droplets on a flat surface. In this system, electrical or acoustic actuation is needed to independently move droplets on the flat surface without the requirement to use pumps. This method has several limitations such as surface fouling, droplet volume restrictions, and difficulties in assembling complex control electronics [72,73]. Conversely, in segmented-flow microfluidic devices, the sample phase is constantly interrupted by an immiscible phase and split into droplets. Several methods for droplet manipulation are available, such as dilution, mixing, merging, splitting, and incubation. The segmented-flow setup overcomes some limitations associated with digital and continuous flow microfluidics, such as fouling and residence time distribution [74,75].

Multiple reactions can proceed within the droplets by altering reaction conditions such as concentration, temperature, and catalyst. Droplet-based carriers can be maintained stably for a long time in the microchannels without fluid evaporation and can be transported whenever needed for further analysis [76]. Automated and computer-controlled microfluidics have been fabricated to control the size, shape, and composition of a gene or drug-loaded droplets. These microfluidic platforms allow rapid single-step or multi-step carrier formation, reduce process variations and improve flexibility in the generation of carriers and drug encapsulation efficiency [22]. A useful programmable valve-actuated microfluidic device has been developed to generate anisotropic elongated particles of the same length, variable bonding angles, pre-designable size sequence and chemical order [77].

Droplet-based microfluidics is commonly used to generate micro-carriers that offer several advantages, including high drug loading dose and sustained drug release for relatively long periods. However, drug release profiles and recognition by the immune system are affected mainly by the surface chemistry of the prepared carriers. Adjusting drug release can be achieved by modifying the internal structure of the droplets by forming double or multiple vesicular layers, which prompt delivery of multiple therapeutic agents. Indeed, coating the fabricated carriers with, for example, poly-(ethylene glycerol) (PEG) can extend the systemic particle circulation and delay cell uptake and clearance [78]. Lipid/polymer microparticles, microcapsules, microbubbles, and microgels can be generated using the droplet-based microfluidic technique through different methods such as a co-flow dripping configuration, evaporation, and extraction [79,80,81,82]. These particles differ mainly in their structure, either solid/gel matrix particles or composed of an inner core and outer shell. Here is the definition for each particle type. Microparticles are spherical solid particles prepared by natural or synthetic materials [83]. Microcapsules are particles that release encapsulated drugs when the outer shell is broken, dissolved, or melted [84]. Microbubbles are gas-filled bubbles that encapsulate a therapeutic agent and burst at a specific site in response to ultrasound [85]. Microgels are polymeric particles that are hydrogels typically composed of water soluble/swellable polymers [86]. Although droplet-based microfluidic devices can generate size-controlled particles, monodispersed carriers are not always obtained due to the subsequent solvent evaporation and droplet solidification step. Moreover, mechanical stirring can disrupt droplet shape, morphology, size uniformity, and loading efficiency [87].

Another disadvantage of droplet-based microfluidic systems is their limitations in generating nano-sized carriers. Recently, there have been several attempts to develop nano-carriers. For example, conjugated polymer nanoparticles (CPNs) with diameters of 256 ± 26 nm were synthesized in a droplet-based microreactor [88]. Other disadvantages of this system are the complexity of handling and the optimization of the fluidic circuit, so their exploitation at an industrial scale is not yet feasible [22].

The most common spherical lipid particles that are formed based on self-assembly or droplet-based microfluidics are emulsions and liposomes, which are discussed in the following section. The choice of mono- or bilayer determines the fluidity or rigidity of the generated particles and influences bioavailability and drug release.

**Figure 3:**
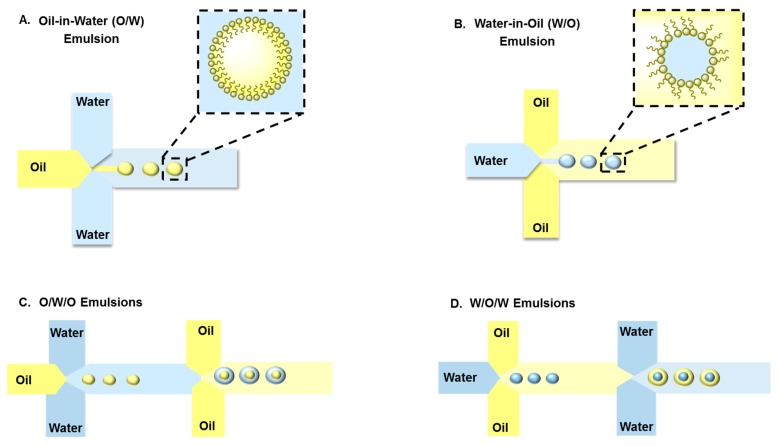
Schematic illustration of microfluidic platforms used to fabricate single (**A**) oil in water (O/W) and (**B**) water in oil (W/O) emulsions or double (**C**) oil in water in oil (O/W/O) and (**D**) water in oil in water (W/O/W) emulsions.

**Emulsions:** Typical emulsions are spherical and form spontaneously from two unmixable solutions where one liquid (the dispersed phase) is dispersed in another liquid solution (the continuous phase) with the help of a suitable surfactant (Figure 3). To enhance long-term emulsion stability and minimize droplet coalescence, a surfactant is used to prepare emulsions. The two main types of emulsions are O/W, in which the hydrophilic head of the lipid/surfactant faces the aqueous exterior and the hydrophobic (or lipophilic) tail faces the inner core, and W/O emulsions, in which the hydrophilic head groups face toward the interior phase and the hydrophobic tail groups of the lipid/surfactant face toward the exterior oil phase [89,90]. Therefore, emulsions can encapsulate hydrophilic and lipophilic molecules such as vitamins, anti-oxidants, and antimicrobials. Several physical and chemical factors affect the size and shape of emulsions such as the pH, temperature, mixing time, viscoelastic shear [91], surfactant concentration, and molecular geometry of the surfactants. There are general methods to prepare emulsions, including phase inversion [92], continental and dry gum, wet gum [93], auxiliary [94], and membrane emulsification [95]. Most of these conventional methods use shear or impact stresses created by manual or mechanical agitation to generate highly polydispersed emulsions. In contrast to traditional bulk emulsification, microfluidic devices are presented as alternative and versatile tools to generate uniform and size-controlled droplets. Moreover, a significant feature offered by microfluidic techniques is the ability to generate single, double, or multiple emulsions. The size and number of the encapsulated droplets can be precisely manipulated by controlling the flow rate of the dispersed and continuous phases [96,97].

The main types of droplet microfluidics that generate emulsions are: (i) continuous-flow-based droplet microfluidics, which result in an emulsion generated from two immiscible fluids; and (ii) electrowetting-based droplets, where an electrical field changes the interfacial tension between the liquid and the surface, producing a liquid finger and then breaking off from the reservoir to generate a droplet [45]. The formation of single, double or multiple emulsion-based carriers by droplet-based microfluidic techniques can be achieved by combining cross-flow, flow-focusing, and co-flow configurations. In a cross-flow microfluidic device, droplets form in a dripping regime with an X-junction, Y-junction, or T-junction. In flow-focusing and co-flow microfluidic devices, droplets form using jetting and dripping modes [54,96,98]. Wang et al. [98] have shown that controlling the flow rate and the solvent diffusion rate have an impact on changing the spherical shape of emulsions generated by microfluidics to a toroidal shape.

Emulsions are extensively used in several applications such as food processing, oil recovery, textile products, and the cosmetic and pharmaceutical industries. Teh et al., [99] reported that a monodispersed double emulsion was formed by flow-focusing channel geometries and solvent exchange. First, single water droplet emulsions were formed in a mixture of 1,2-dioleoyl-sn-glycerol-3-phosphocholine (DOPC) into oleic acid. Then, the W/O emulsion stream was sheared into a double emulsion at the second junction by another aqueous solution consisting of a mixture of ethanol, glycerol, and Pluronic surfactant in water. The produced particles are stable, have a shelf-life of approximately three months, and monodisperse in sizes with less than 2% variation. A combination of DOPC lipid vesicles with porous silicon (PSi) micro-particles was generated as a multistage drug delivery system using a glass-capillary flow-focusing microfluidic device. Thermally hydrocarbonized PSi particles were encapsulated into the aqueous core of W/O/W emulsions with ultrathin shells. The resulting particles have high loading capacity for hydrophobic drugs and sustained release due to their synergistic effect [100]. Pessi et al., [101] formed polymeric microcapsules using a microfluidic device that employs a biphasic flow to generate W/O/W double emulsion droplets containing bovine serum albumin (BSA) as a template for a therapeutic protein. The generated microcapsules were monodispersed with low porosity and high stability. The most important feature of these particles is the high protein encapsulation efficiency, which reached approximately 84%.

The main disadvantages of emulsions are they are thermodynamically unstable, short shelf-life, improper selection of surfactants causes phase inversion while improper formulation causes creaming or coalescence of emulsions. Furthermore, microfluidic technology usually generates large emulsion particles which is unfavorable for most applications.

**Figure 4:**
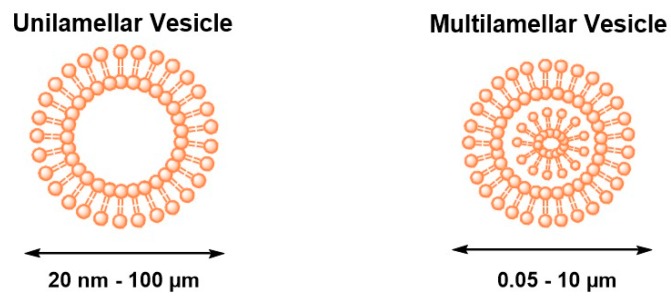
Schematic drawing of two different types of liposomes.

**Liposomes:** Liposomes are amphiphilic lipid vesicles that are considered one of the most successful candidates for drug delivery due to their lipid arrangement, which mimics the biological cell membrane. Their size and dimensions can vary from tens of nanometers to several hundred micrometers, and they can be composed of a single closed shell or more (unilamellar or multilamellar vesicles, respectively) (Figure 4). Liposomes are formed spontaneously in aqueous media into spherical bilayers with an aqueous interior core surrounded by a hydrophobic lipid bilayer membrane [102,103]. Hence, liposomes can be loaded with hydrophilic or hydrophobic molecules. Hydrophilic agents can be entrapped in the interior core, while hydrophobic agents can be solubilized or inserted into the lipid membrane. Membrane proteins can also reconstitute into the liposome membrane to retain their activity. There are several techniques to generate liposomes, including lipid film hydration, sonication, extrusion, reverse-phase evaporation, and high-pressure homogenization. Preparation methods can influence liposome size, lamellarity, and encapsulation efficiency. Recently, the development of microfluidic devices helps in the generation of monodispersed liposomes by decreasing the time and the number of steps [104]. The droplet-based microfluidic system is one of the most common microfluidic approaches employed in liposome production [50]. This strategy is based on manipulation of two immiscible phases that generate micrometer- to sub-micrometer-sized droplets (usually W/O emulsions). The nanoparticles (i.e., liposomes) then form in the droplets. Martz et al., [105] designed a flow-focusing device with a pressure-controlled system to generate lipid vesicles with a diameter less than 100 nm. By controlling the pressure and the flow rates of the continuous and dispersed phases, micro- and nano-droplets were produced for acoustic droplet vaporization applications. Davies et al., [106] reported the development of a 3D flow-focusing microfluidic device modified with coronal discharge. In this system, after solvent extraction and, by controlling the capillary numbers of immiscible flows, the generated double emulsion can be transformed into liposomes.

Another simple microfluidic strategy for liposome generation is the continuous flow system, which uses microfluidic hydrodynamic focusing (MHF) to synthesize liposomes with particle sizes ranging from 50 to 500 nm. This strategy is based on the improvement of the classical alcohol injection method and was first reported by Jahn et al., who developed a microfluidic chip with four-microchannel intersection geometry and an organic solvent with dispersed lipids. Into the middle stream, the lipid solution was injected and compressed by two fluid streams. The monitoring of the FRR between buffers and organic fluids and the total volumetric flow rate (Q_t_) in the outlet stream enables aqueous and organic solutions to mix through molecular diffusion, leading to phospholipid self-assembly into liposomes [107,108]. In a continuous flow process, the steps of monodispersed liposome production, buffer exchange, and liposome/drug mixing and incubation are all performed in a microfluidic device. Hood et al., [109] have reported a rapid and efficient remote loading of amphipathic drugs into liposomes in a continuous integrated system. The developed microfluidic device was able to reduce the processing time for liposome formation and remote drug loading from several days to less than three minutes with a five times higher drug-to-lipid ratio (D/L) compared to drug loaded-liposomes prepared by conventional methods [110].

The design of the microfluidic chip has a significant impact on liposome size, which strongly affects their cellular uptake. Andar et al., [111] synthesized self-assembled monodispersed liposomes by the HFF method and investigated the effect of size on cellular uptake mechanisms. Small liposomes follow dynamin-dependent endocytosis, while large liposomes follow clathrin-dependent endocytosis when these particles were tested against endocytosis inhibitors. Liposomes are widely used in the cosmetic, pharmaceutical, food and farming industries. Many biologically active (unstable) compounds, including anti-cancer and anti-microbial agents, peptides/proteins, vaccines, and genetic materials, have been successfully trapped into liposomes [112]. For example, in the field of gene therapy, cationic liposomes are considered as promising gene delivery vehicles due to the ability of cationic lipids to form electrostatic complexes with DNA, which can then be delivered to the cell nucleus [113,114].

Liposomes also suffer from some limitations including low encapsulation efficiency in some cases, cytotoxicity of some cationic formulations, and physicochemically instability of some formulations. Similar to emulsions, the formation of liposomes in the nano-scale using microfluidic devices is not easy to achieve and requires highly controlled conditions.

## 4. Non-Spherical Drug Delivery Systems

**Figure 5:**
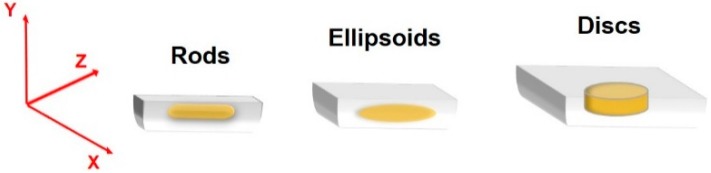
Schematic drawing of different types of non-spherical particles. Designing masks with different channel heights resulting in particles with different shapes, sizes and aspects ratios [123,124].

Although it is more common to generate spherical particles by the microfluidic technique, non-spherical particles can also be generated as drug carriers due to their ability to mimic natural structural and functional properties of the biological cells (Figure 5). There are several strategies to form non-spherical delivery systems in different sizes and shapes by microfluidic channels, including self-assembly of spherical building blocks, stretching and deforming droplets before or during solidification in the confined channels, or by flow lithography [98,115,116]. For example, droplets can be confined to micro-channels, and the deformation results in the generation of disks, rods, or ellipsoids if the droplet volume is larger than the largest sphere accommodated in the channel [117,118]. The importance of these particle shapes is their role in in vivo biodistribution, circulation time in the blood, and cellular uptake mechanisms [119]. In one study, filomicelles with paclitaxel were developed and investigated in mice. The long-circulating time of these drug delivery systems increased the apoptosis rate of the cancer cells and reduced the tumor size [120]. Several studies showed that anisotropic nanoparticles can avoid bioelimination more efficiently than spherical nanoparticles under the same conditions [121]. Kolhar et al. [122] showed that gene silencing efficiency was enhanced in the vascular endothelium by delivering siRNA using needle-shaped polymeric nanoparticles compared with spherical particles. 

## 5. Nucleic Acid Delivery Systems

Microfluidic techniques for gene carrier fabrication and DNA synthesis have been developed using lower reagent volumes, to make the process more cost-effective. Cationic polymers and lipids are the major components that are used to transfer materials of synthetic genes. Polyplexes, lipoplexes, or lipopolyplexes can be formed by mixing anionic nucleic acids with cationic polymers, lipids, or both, respectively [18]. Several factors such as the reagent concentrations, solvent mixing order, and speed affect the size, charge, and shape of the resulting particles. Polydispersed synthetic nucleic acid particles are commonly formed when formulated in bulk, while diffusion-based microfluidics can form monodispersed particles. In one study, polyethylenimine (PEI)-plasmid DNA (pDNA) nano-carriers were developed using hydrodynamic focusing and a microfluidic device. The resulting gene carriers had a highly uniform particle size distribution compared to the bulk preparation. These carriers enhanced cell viability and gene transfection efficiency [125]. In another model using a mixture of polymer and lipids, lipopolyplexes were created in two steps to obtain a lipid membrane encapsulating a core composed of poly(L)lysine (PLL)-pDNA polyplexes. First, a polyplex core was formed by injecting PLL and pDNA into the microfluidic channels. Second, lipids and the polyplex were mixed in the device to generate a lipid-encapsulated polyplex [126]. Balbino et al. [127] reported on the generation of high-yield cationic liposomes using two techniques, single hydrodynamic focusing (SHF) and double hydrodynamic focusing (DHF). The techniques were used to form particles with diameters of 100–130 nm and to assess the effect of several parameters such as total lipid concentrations, FRR, and average fluid flow velocities on droplet generation. The transfection ability of formed unilamellar liposomes was tested in human epithelial carcinoma cells (HeLa), and the results confirm the ability of these carriers to be used for gene delivery and vaccine therapy [128].

T-junction and flow-focusing configurations are also used to generate nucleic acid carriers by a microfluidic droplet generator. Nanocarriers generated by the droplet technique are usually used to prompt minimal apoptosis in cells. In one study, the nanocarriers formed by a microfluidic device showed a continuous uptake profile for approximately 5 h, with 90% cell viability and 60% transfection efficiency, while the uptake reached plateau after approximately 2 h of incubation. with the nanocarriers formed by the bulk method. The particles formed by the bulk method had 75% and 40% cell viability and transfection efficiency, respectively [18].

For rapid DNA hybridization assays, a compact disk microfluidic device was fabricated based on reciprocating flow with nanoliter volumes. The Dengue virus gene sequence was used to test the model. The reciprocating flow of DNA samples improved the mass transfer rate and decreased the hybridization time to 90 s [129].

Generation of nucleic acid delivery systems for clinical application is still a challenging task because nucleic acids are usually unstable. Although microfluidics improves the quality of nucleic acids delivery systems, scaling-up these devices remains difficult.

## 6. Application of Microfluidics for Protein Synthesis and Drug Screening

The following section describes the utilization of microfluidic technologies for protein synthesis and for fabrication of in vivo mimicking systems that allow high-throughput drug screening and the development of devices for use at the point-of-care (POC). 

### 6.1. Cell-Free Protein Synthesis on a Chip

**Figure 6:**
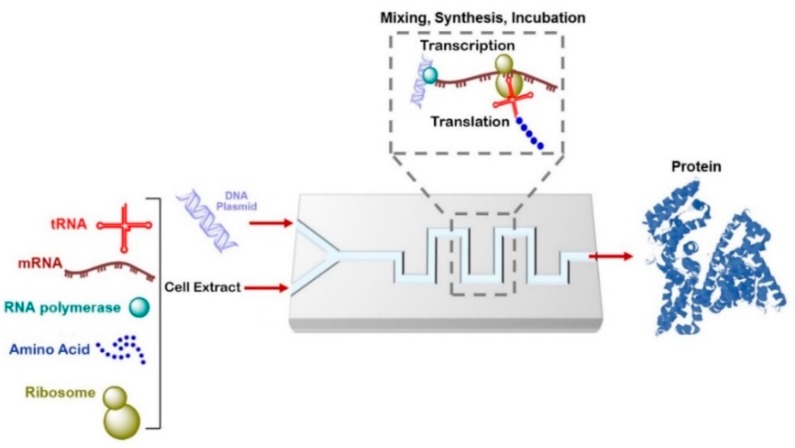
Schematic drawing of cell-free protein synthesis in a microfluidic device. tRNA: transport RNA; mRNA: messenger RNA.

The production of therapeutic proteins by genetic engineering is attracting more attention with the rapid developments in biotechnology. Natural or genetically modified proteins can be used as active substances for therapeutic uses. Several pharmaceutical proteins are difficult to express or are toxic to living cells. As a promising alternative to cell-based protein synthesis, pharmacologically relevant proteins can be synthesized efficiently with cell-free systems. Cell-free protein synthesis (CFPS) is simple, fast, reduces the required sample/reagent volume, controls and adjusts the reaction conditions easily, and removes the need for cell transformation or culturing, thereby accelerating the screening of large protein libraries (Figure 6). Integration of CFPS in a microfluidic device has many advantages, including achieving high-throughput protein expression, stimulating the expression of multiple proteins, reducing reagent consumption (which reduces the cost of protein synthesis), enabling the production of a single-dose of therapeutic proteins, allowing rapid protein detection and analysis, and eliminating the requirement to harvest proteins [130,131,132,133,134]. Mei et al., [135] reported the fabrication of a microfluidic array device for continuous exchange. This device comprises a tray and a wall chamber separated by a dialysis membrane for CFPS and a nutrient reservoir. The green fluorescent proteins chloramphenicol acetyl-transferase and luciferase were synthesized in this microfluidic device. Although the protein production is 5–10 times longer than the production in a micro tube, the production yield was 13–22 times higher. In another study, a microfluidic 96-well plate with continuous cell-free protein expression components had a production yield up to 87 times higher than a traditional batch system [136]. 

A droplet-based microfluidic system has also been exploited for in vitro protein synthesis in droplets by combining on-chip and off-chip operations. Translation and transcription of the *Bacillus subtilis cotA laccase* gene and subsequent kinetic analysis of the synthesized protein have been investigated in the developed system [137]. Another use of Droplet-based microfluidics is to amplify single genes by droplet-based PCR and then to translate the amplified gene into protein using a cell-free system [138]. In general, the applications of microfluidic devices are still limited for clinical diagnosis at the POC. In one study, CFPS has been integrated with a microfluidic bioreactor and optimized for POC use. The fabricated dual-channel bioreactor allowed the exchange of energy, metabolites, and inhibitors between the reactor and feeder channels and higher synthesis of a single-dose therapeutic protein than the traditional tube-based batch system [139].

### 6.2. Cell-on-a-Chip

**Figure 7:**
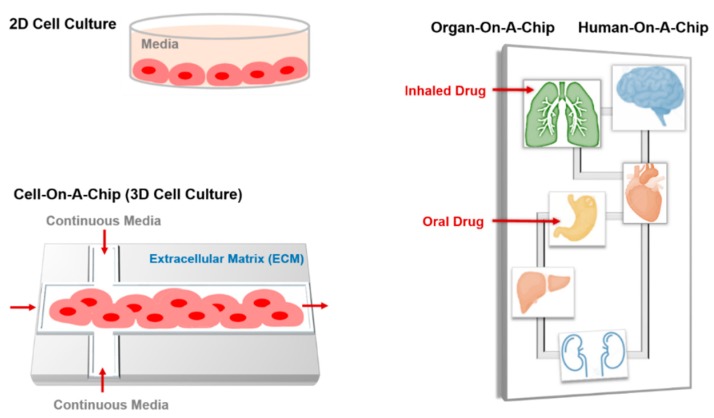
Schematic drawing of 2D cell culture, cell-, organ-, and human-on-a-chip.

Two-dimensional cell culture is routinely undertaken in all laboratories worldwide to simplify the extracellular matrix (ECM) and cell processes, for the purpose of gene expression, drug delivery, apoptosis, and toxicology studies. However, it is still difficult to completely mimic the in vivo system and reflect their anatomic and physiologic properties. Here, microfluidic technology offers new opportunities for cell-based sensors and multifunctional platforms for biochemical and biomedical functions under physiologically relevant conditions (Figure 7). In microfluidic devices, integrating surfaces that mimic the biochemistry and geometry of ECM with microfluidic channels enables the control of cell growth and stimuli. These devices allow cell growth in three dimensions in ECMs such as hydrogels, droplets, and scaffolds. The 3D culture improves tissue organization; enhances the expression of differentiated functions; and allows high-resolution live imaging and high-throughput and low-cost drug screening by investigating the effects or cytotoxicity at multiple doses in parallel, thereby saving reagents, time and labor.

Cancer cells differ significantly in their response to therapy, development of drug tolerance, survival, and metastatic potential [140]. Therefore, the screening for potential drug combinations is a challenging task, requiring minimizing the screening cost, since the consumption of samples and reagents for combination drug screening experiments is much higher than that for single drug screening. Indeed, to be relevant for modeling in vivo behavior, it needs to be performed at least at the cell level [141]. A droplet-based microfluidic system provides a high-throughput assay for investigating potential drug combinations due to its low cost and high sensitivity by generating thousands of droplets with different concentrations, resulting in a chemical library used to define a dose-response curve at a significantly high resolution [142,143,144]. This system lowers sample and reagent consumption from the microliter to the nanoliter range and minimizes the multistep liquid handling operation [145]. Combining microfluidic chips with fluorescence microscopy allows the analysis of cell viability with single-cell resolution in a suitable manner. Indeed, droplet microfluidics can compartmentalize single cells, thus allowing the investigation of cytotoxic drug uptake and apoptosis without affecting the functionality of adjacent cells [146].

A microfluidic cell culture was integrated with a commercial 96-well plate without any requirements for tubing or pumping, which maintains long-term continuous perfusion cell culture and allows direct analysis of the wells of the microfluidic plate. In this system, HeLa cells were cultured in the microfluidic device, and the cytotoxicity effect of the anticancer drug etoposide was tested using the lactate dehydrogenase (LDH) assay. Further development of this device will enable automated cell-based screens, improve the potential for high-throughput assays, increase data quality, and reduce cost [147].

One of the best in vitro models for cancer is a tumor spheroid, which comprises a gel-free system. A microfluidic device was developed by incorporating microwalls that induced the seeded human carcinoma cells HT-29 to aggregate and create spheroids spontaneously. Subsequently, the formed spheroids were exposed to multiple doses of 5-fluorouracil, and cell viability was investigated by measuring spheroid size. The created spheroids showed high viability for 10–12 days, which suggests this culture system is a promising platform to study drug effects in the long-term analysis [148].

In many 3D cell culture systems, the cells are seeded within a hydrogel network that acts as a scaffold or matrix while permitting oxygen diffusion and nutrient transportation. Several gel-supported 3D cell cultures in microfluidic devices have been designed using native ECMs such as agarose, collagen, hyaluronic acid, fibronectin, and fibrin [10,149,150]. In one study, a combination of collagen and hyaluronic acid in a microfluidic channel was used to mimic sprouting angiogenesis in vitro to study endothelial cell sprouting and migration. The combination improved adhesion, migration, and proliferation of human umbilical vein endothelial cells (HUVECs) in response to the vascular endothelial growth factor (VEGF) [151]. On the other hand, doxorubicin was perfused through a microfluidic channel containing breast cancer cells (Her-2) encapsulated within an alginate hydrogel and cultured into a 3D tumor spheroid. Compared to 2D cell culture, doxorubicin had lower effectiveness on cell viability and proliferation [152].

Although 3D cell cultures have direct applications in drug screening, tissue engineering, and regenerative medicine, this system suffers from several limitations. For example, in the hydrogel-supported 3D cell cultures, the gel matrix may prevent the cells from culturing at a high enough density, resulting in the formation of interstitial spaces. Additional research in this field is required to optimize the system. To date, the best developed 3D culture systems failed to mimic the full in vivo tumor model since vascularization during tumor development is not taken into consideration. 

### 6.3. Organ-on-a-Chip

Although there are advantages to 2D cell culture, this system suffers from some limitations, such as the restricted simulation of the complex cell-cell and cell-matrix interactions to investigate cell behavior. In the organ-on-a-chip microfluidic devices, living cells can be cultured in a continuously perfused micro-chamber to pattern the physiological functions of tissues and organs [153]. The resulting devices incorporate functional units with a minimal number of living components of an organ that sufficiently retains the tissue- and organ-level interactions. Depending on the tissue perfusion and micro-architecture, fabricated devices can last for approximately one month, which enables the study of biological phenomena and underlying mechanisms of organ physiology [154]. The organ-on-a-chip is a fast-developing area, as evident from the special issue dedicated to the topic in the Genes journal [155].

Concerning the biological barriers such as the blood-brain barrier (BBB), the lung alveolar-capillary interface, and the kidney transport barrier, multilayered membrane-based microfluidic devices mimicking biological systems were developed to examine drug effects on different organs. 

Lung-on-a-chip devices are widely used to examine the toxicity of several nanoparticles and to understand the pulmonary diseases resulting from blockage of the small airways due to liquid plug formation that prevents gas flow in alveoli [156]. Oxygen transfer efficiency and fluid mechanical resistance are two critical parameters that must be taken into consideration during the design of the human lung [157]. Unlike most organs, the epithelial cells in the lung are exposed to the air, which makes this layer susceptible to pathogens and air pollution. Therefore, many microfluidic devices were fabricated that simulate air–liquid and air–cell interfaces to study the cellular flux of metabolites, drugs, and small toxic molecules. In one fabricated microfluidic device that mimics the breathing model, two chambers are built, and the air is pumped continuously at certain pressures, imitating inhalation and exhalation patterns. In the central chamber, a thin, flexible PDMS layer was used to separate cultured human alveolar epithelial cells and cells of the blood vessel wall. This membrane is stretched and relaxed according to the air flux. This lung-on-a-chip device was used to test the toxicity and inflammatory effects of silica nanoparticles on the lung epithelium [158]. The mechanical strain of the membrane improved the epithelial and endothelial uptake of the nanoparticles, which presents the fabricated device as a highly accurate and low cost in vitro model for clinical studies for drug screening and toxicology. In another in vitro model developed by Douville et al., [159], two different alveolar epithelial cells (transformed human alveolar epithelial cell line (A549) and primary murine alveolar epithelial cells (AECs)) separated by a PDMS thin membrane were exposed to a combination of surface tension and solid mechanical stresses. Significant morphological differences between these two alveolar cell types have been reported, as well as significant differences in cell death and cell detachment rates, which may help in developing techniques to minimize and eliminate fluid stress in clinical studies.

To screen or investigate the toxicity of drugs on the kidney, a kidney-on-a-chip system was designed by mimicking renal cells or nephrons on a microfluidic device to study filtration and reabsorption of drugs and their induced damage that may cause cancer or acute tubular necrosis [160]. An in vivo-like tubular environment was developed by designing a multi-layered microfluidic device composed of integrating a PDMS microfluidic channel and a porous membrane to resemble the renal tubule system and using the outer tubular fluid and a continuous stream of inner tubular fluid as analogs for blood and urine precursor fluid, respectively. In this device, rat inner medullary collecting duct cells were cultured, and a fluidic shear stress was applied. It was reported that hormonal stimulation allows water-transporting proteins to move within the cells similarly to the native tissue, which regulates water and ion balance through molecular transport [161].

The liver is one of the most complicated organs; it is responsible for thousands of functions in biological systems and plays a crucial role in the investigation of drug interactions. Hence, the fabrication of liver-on-a-chip devices aims to study pharmacokinetic parameters such as hepatic clearance, bioavailability, and cell permeability. Lee et al., [162] reported the development of a microscale primary rat and human hepatocyte culture chip that allows cell-cell interaction and defines a tissue and fluid transport region. Continuous nutrient exchange maintained cell viability for more than seven days. Another hepatic model cultured on a hydrogel layer enabled analysis of drug hepatotoxicity in combination with a mathematical pharmacokinetics–pharmacodynamics model [163]. A multi-walled liver microfluidic device has assessed the gene expression profile, phase I/II metabolism, secretion and transport of liver products, and susceptibility to hepatotoxins [164]. These fabricated microfluidic devices aid in the investigation of liver function and hepatotoxicity in a short-time, are not labor-intensive and are less expensive than animal studies.

Another organ that has been modeled on microfluidic devices is the heart. The heart-on-chip system allows the investigation of contractility and electrophysiological behaviors under in vitro conditions. Moreover, it allows quantification of cardiac tissue contractility under various conditions of health, disease, and exposure to chemical agents [18]. To study structure–function relationships in the human heart, a biohybrid model was constructed based on muscle thin film (MTF) of a cardiac tissue engineered myocardium composed of anisotropic cardiomyocytes cultured on elastic thin films. The developed chip allowed quantification of contractile stress as a function of time, electrophysiology, and inter-and intracellular architecture [165].

Simulation of the in vitro gut environment is accompanied by several difficulties, such as allowing the growth of the normal flora, mimicking the mechanically active environment, and providing sufficient density of intestinal villi [18]. Similar to the lung-on-a-chip model described previously, a human gut-on-a-chip model consists of two micro-channels separated by a porous flexible membrane coated with ECM and lined by human intestinal epithelial cells (Caco-2). A slow fluid flow generated low shear stress over the channels, and a cyclic strain was exerted to mimic physiological peristaltic motions. The developed model allowed spontaneous growth of 3D villi-like structures and quantified the trans-epithelial electrical resistance (TEER), which was three- to fourfold higher than that of static Transwell cultures. Two important observations were noticed in this study. First, as time increased, TEER values improved, and second, the media dilution rate was induced by continuous perfusion, which supports long-term co-culture of both epithelial and microbial cells [166].

The BBB-on-a-chip model mimics the structure and physiological complexity of the human BBB and allows investigation of central nervous system diseases. A dynamic neonatal BBB-on-a-chip was developed by co-culturing neonatal rat brain endothelial cells and astrocytes under shear flow that allows endothelial cell and brain interactions. The designed model was flexible and allowed direct monitoring of dynamic processes in real-time, and the permeability was not significantly different from that of in vivo BBB permeability in neonatal rats [167].

All previously discussed biomimetic organ-on-a-chip microfluidic devices present several advantages in accuracy, scalability, low cost, and high throughput in mimicking human organs, allowing reproducible drug screening, while reducing the number of animals used prior to clinical studies.

### 6.4. Human-On-A-Chip

This model envisions moving beyond individual tissue and organ engineering to study drug metabolism, toxicity, accumulation, and distribution at the level of the whole organism. Human-on-a-chip microfluidic devices are composed of interconnected chambers, each representing a specific organ by containing one cell type, connected by a microfluidic circulatory system [168]. One microfluidic device model composed of 2D cultures of liver and lung cells was developed successfully as a pharmacokinetic model [169]. Another multi-organ device comprising four different cells representing the lung, kidney, liver, and lipid tissues was developed by Zhang et al., [170]. Each cell type was cultured in a separate compartment, and transforming growth factor β1 was released to maintain the functionality of different cell types. The media were perfused to the center from the two-way channels and subsequently passed to the other channels after perfusion through the lung. Human-on-a-chip is useful, as the drug targets the specific organ, but also affects other organs in the system. The Human-on-a-chip allows checking the toxicity of the drug in various organs, at near physiological conditions.

## 7. Current and Future Perspectives

**Figure 8:**
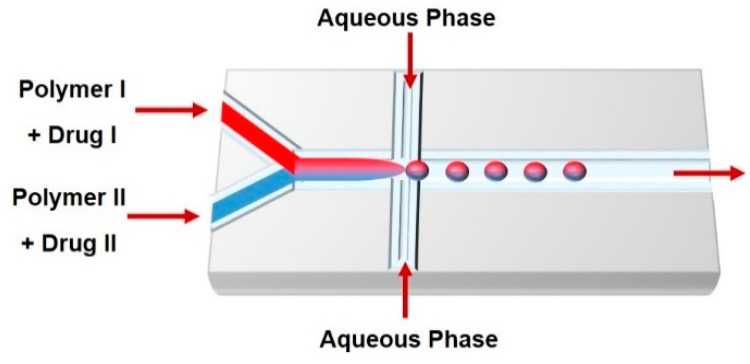
Schematic drawing of generation of Janus particles based on microfluidic chip.

Currently, there is escalating effort in the development of microfluidic devices since these devices are equivalent or superior to several existing alternatives. Microfluidics have great potential in generating micro- and nanoparticles as promising drug delivery systems [171]. Generation of microparticles or nanoparticles as drug delivery carriers using microfluidics offers several advantages such as miniaturization of the fluidic environment, efficient utilization of materials and excipients, and precise control of particle specifications and performance metrics [172,173]. One example of exploiting microfluidic platform to produce smart drug delivery system is the generation of Janus particles (Figure 8). Janus droplets are spherical particles with chemically and/or physically distinct segments. This unique feature allows two different reactions to occur on the same particle. Janus particles can potentially be used to deliver multiple agents with different solubilities [174,175,176]. Xie et al., [175] fabricated a fluidic nanoprecipitation system that enables one-step generation of Janus polymeric nanoparticles composed of poly(lactic-*co*-glycolic acid) (PLGA) to encapsulate paclitaxel and doxorubicin hydrochloride (hydrophobic and hydrophilic drugs, respectively) on different sides of the particle. Another model developed by Lone et al., [176] is a T-junction microfluidic chip, wherein Janus particles were synthesized by UV-directed phase separation in the downstream channel. The formed particles are photo-curable W/O emulsions employing a light-sensitive copolymer. 

In the pharmaceutical industry, unsustainable R&D costs hinder development and approval of new drugs [177,178]. This obstacle can be solved by using microfluidic devices either to synthesize particles for drug delivery or to screen newly developed drugs. Microfluidic platforms suffer mainly from the limited quantity of synthesized particles. Such challenge can be overcome by parallel arrangement of several micro-channels on a single chip to increase the production rate up to few kilograms per day, which is appropriate for industrial-scale production [179,180]. Moreover, the next-generation technique for the pharmaceutical application can be the microfluidic cell culture. Currently, 3D bioprinting is allowing the design of several organ-on-a-chip platforms [181]. Mimicking in vivo conditions of normal or disease states on a single in vitro device is expected to enable investigation of drug toxicity, efficacy, and pharmacokinetics [182]. Such biomimetic systems may improve the predictability of the effects of a new therapeutic agent before animal testing or may offer a superior alternative to animal studies [183]. The future of bioprinting technology includes the 4D systems, which aim at the creation of dynamic 3D patterned biological structures. The suggested model exploits several stimuli-responsive biomaterials (e.g., bioink), and the fabricated biological construct can potentially transform its shape and behavior in response to various stimuli [184].

## 8. Conclusions

Remarkable developments in the fabrication of microfluidic devices have occurred during recent decades, either to generate effective drug delivery systems or to improve screening strategies. Micro- or nanoparticles produced by microfluidic systems are uniform in size and morphology, encapsulate more agents, and exhibit slower drug release rates than some formed by bulk methods. The physical and chemical features of drug carriers can be modified by controlling the flow rates of aqueous solutions, composition, temperature, pH, and other environmental factors. The main obstacle facing any drug delivery system is how to achieve long-term stability in the shelf and in bodily fluids under biological conditions. Hence, more efforts are still needed to improve microfluidic platforms to generate and analyze particles or carriers with higher stability and biocompatibility. Replication and mimicking of in vivo conditions through microfluidic platforms can enhance drug cytotoxicity screening and allow for inexpensive partial alternatives to the animal models. Continuous discovery and development in tissue engineering enhance understanding and investigation of new drug effects in clinically relevant microfluidic high-throughput platforms. Cell-on-a-chip, organ-on-a-chip, and human-on-a-chip microfluidic devices for drug screening have excellent potential for use at the point of care.
